# Evaluation of novel 3D-printed and conventional thermoplastic stereotactic high-precision patient fixation masks for radiotherapy

**DOI:** 10.1007/s00066-022-01963-w

**Published:** 2022-06-13

**Authors:** Veronika M. Miron, Tanja Etzelstorfer, Raimund Kleiser, Tobias Raffelsberger, Zoltan Major, Hans Geinitz

**Affiliations:** 1 Institute of Polymer Product Engineering, Johannes Kepler University, Altenberger Str. 69, 4040 Linz, Austria; 2 Abteilung für Radioonkologie, Ordensklinikum Linz Barmherzige Schwestern, Seilerstätte 4, 4010 Linz, Austria; 3 Department of Neuroradiology, Johannes Kepler University Clinic, Wagner-Jauregg-Weg 15, 4020 Linz, Austria

**Keywords:** Additive manufacturing, 3D printing, Patient fixation masks, Radiotherapy

## Abstract

**Purpose:**

For stereotactic radiation therapy of intracranial malignancies, a patient’s head needs to be immobilized with high accuracy. Fixation devices such as invasive stereotactic head frames or non-invasive thermoplastic mask systems are often used. However, especially stereotactic high-precision masks often cause discomfort for patients due to a long manufacturing time during which the patient is required to lie still and because the face is covered, including the mouth, nose, eyes, and ears. To avoid these issues, the target was to develop a non-invasive 3D-printable mask system with at least the accuracy of the high-precision masks, for producing masks which can be manufactured in the absence of patients and which allow the eyes, mouth, and nose to be uncovered during therapy.

**Methods:**

For four volunteers, a personalized 3D-printed mask based on magnetic resonance imaging (MRI) data was designed and manufactured using fused filament fabrication (FFF). Additionally, for each of the volunteers, a conventional thermoplastic stereotactic high-precision mask from Brainlab AG (Munich, Germany) was fabricated. The intra-fractional fixation accuracy for each mask and volunteer was evaluated using the motion-correction algorithm of functional MRI measurements with and without guided motion.

**Results:**

The average values for the translations and rotations of the volunteers’ heads lie in the range between ±1 mm and ±1° for both masks. Interestingly, the standard deviations and the relative and absolute 3D displacements are lower for the 3D-printed masks compared to the Brainlab masks.

**Conclusion:**

It could be shown that the intra-fractional fixation accuracy of the 3D-printed masks was higher than for the conventional stereotactic high-precision masks.

## Introduction

The diagnosis of intracranial malignant or benign tumors can be particularly shocking and lifechanging because these tumors can affect the patients’ thought processes, their motor function, or their senses. A treatment method especially suitable for the treatment of non-bulky brain metastases or benign tumors like cranial nerve schwannomas is either radiosurgery or fractionated stereotactic radiation therapy. The aim of these techniques is to target the tumor precisely with high-energy X‑rays or protons in order to destroy the tumor cells without compromising the surrounding healthy tissue. The dose is either given in one fraction (radiosurgery) or divided into several smaller fractions (fractionated stereotactic radiation therapy) given over several days or weeks [1].

For cranial stereotactic radiation therapy, accurate treatment positioning with high repeatability and patient immobilization is important [2]. Several studies have investigated the inter- [1, 3–8] and intra-fractional [2, 5, 7, 9] fixation accuracy of diverse systems. Mean setup errors range from 1 mm for stereotactic head frames [10], to ~ 2 mm for Gill–Thomas–Cosman (GTC) frames [3], and up to > 3 mm for thermoplastic mask systems (e.g., Brainlab mask; Munich, Germany) [3]. Mean intra-fractional motion with GTC frames is given with 0.54 mm [2] and for thermoplastic masks with 0.73 mm [2] up to > 3 mm [5]. However, the achievable accuracy of immobilization and patient positioning is not only dependent on the fixation system used, but also on the compliance of the patient (how long the patient can lie still during the treatment) and the uncertainty from planning with image registration from multiple imaging modalities [11].

Conventional high-precision masks for stereotactic radiation therapy are made from a thermoplastic mesh which is heated in a hot water bath and then thermoformed over a patient’s head. In the case of the latter model, it consists of a mesh supporting the dorsal and occipital parts of the head, a divided plate, and a second mesh for on top of the face (see Fig. 2a). The cooling and subsequent hardening of the mask takes around 45 min. With all preparations, the patient and hospital staff are occupied for around 1 hour in which the patient has their head movement restricted. As mouth, nose, and eyes are covered with the mesh for this type of mask and the material shrinks during cooling, this can raise discomfort for the patient, especially for children or those suffering from claustrophobia [12]. However, other stereotactic masks, especially those used in conjunction with surface scanning, render at least nose and eyes free from mask material [12].

Three-dimensional (3D) printing allows manufacturing of fixation masks in the absence of the patients to spare them the thermoforming process and create the masks directly personalized to the patient’s head. One way of using 3D printers to create fixation masks was suggested by Pham et al. (2018) who used printed head phantoms of patients to thermoform thermoplastic masks for patients with palliative whole-brain radiotherapy [13]. This process would allow use of certified thermoplastic masks and spare patients the mask-molding process. However, the first report of a directly 3D-printed face mask for radiotherapy was already published in 2002 by Sanghera et al. [14]. The idea was to use 3D surface scanning to create a virtual mask that was then 3D printed with fused filament fabrication (FFF), an extrusion-based additive manufacturing technique, using the material acrylonitrile butadiene styrene (ABS). It could be shown that the process is feasible; however, the mask design was a simple offset from the full facial surface without cutouts for eyes and airways or a frame for fixation. The accuracy of the printing process and the attenuation properties of ABS were measured and judged as sufficient; it was, however, pointed out that shrinkage and deformation after production should be studied further [14]. A disadvantage was the long printing time (5 days), which was typical for printers in 2002.

Years later, in 2014, the idea was rediscovered in 3D-printed patient-specific bolus material for electron radiotherapy, and a group in the UK produced a head shell based on CT data tested with a phantom [15]. After evaluating the virtual model with the immobilization system and the 3D-printed model on a phantom in CT, they suggested that 3D-printed masks can achieve similar levels of immobilization to conventional systems [15]. A year later they published an analysis of 3D-printing materials and their dosimetric properties compared to an Orfit thermoplastic mask (Orfit Industries NV, Wijnegem, Belgium). For one healthy volunteer, a head phantom and face shell (without fixation frame) based on MRI data were designed and printed 50% scaled down with a Z-Corps 450 (powder + binder) 3D printer. A full-scale face shell was also successfully printed using VeroWhitePlus^TM^ as material [16].

The first comparison of fixation accuracy between a 3D-printed mask and a conventional mask was reported in a poster by Sato et al. (2016) [17]. They used a patient’s CT data to create a mask and headrest of unspecified design. The mask, headrest, and head were 3D printed with “ABS resin”. With the head phantom, a conventional mask was thermoformed. Each mask was used fifteen times for a set-up and CT images were acquired and compared. The results suggest that 3D-printed masks could show a similar positional accuracy to conventional masks [17]. Because standard Brainlab SRS (stereotactic radiosurgery) headrests were too big for children, Luo et al. (2018) compared 3D-printed headrests with Brainlab SRS standard headrests for pediatric patients and showed that the 3D-printed headrests can reduce setup errors in the ventral direction [18].

In 2016, Chen et al. presented the first automatic process for mask design via automatic segmentation, atlas registration, image analysis, and automatic detection of facial features such as eyes, nose, and mouth. The process was tested on CT data of 10 patients and the resulting design was compared to the CT data that it was constructed from. The addition of a frame for mounting the mask on the table and tests with healthy volunteers or patients were not performed [19]. Haefner et al. [20] followed up by evaluating the setup accuracy of 3D-printed masks for 10 healthy volunteers for semi-automatically designed masks. Even though the design process was not described in detail, the reported results for inter-fractional positioning accuracy are in the range of conventional masks. Asfia et al. (2021) studied topology optimization based on pressure sensors for two different mask designs, “one with a nose bridge and one open as would be used with optical surface guidance” [21].

Depending on the design, 3D-printed fixation masks can improve patient comfort, also due to less necessary patient visits to the hospital and elimination of the thermoforming process to manufacture the masks. A big advantage is also the option to include additional features in the masks, such as markers, bolus, or design features (e.g., Mickey Mouse mask for pediatric patients). According to the abovementioned studies, the setup accuracy for fixation lies in the range of conventional thermoplastic masks, although lower than for invasive fixation. However, the attenuation properties of selected mask materials, speed of design and manufacturing, as well as the costs of 3D-printed masks compared to conventional masks should be further analyzed [22].

For this study, a survey among patients and radiotechnologists was conducted to identify issues with standard and high-precision masks currently used at the hospital *Ordensklinikum Linz Barmherzige Schwestern*. All in all, the information given and the caretaking were satisfactory for the patients; however, the mask-making was considered more problematic/uncomfortable than the radiation therapy itself. This was especially true for patients wearing the stereotactic high-precision mask, which was probably because the making took longer than for the other masks and longer than single radiation fractions. 75% of these patients would have preferred to have avoided the mask-making process altogether. Radiotechnologists reported that the feedback of patients towards them is more positive than negative overall for patients wearing all types of masks, which coincides with the patients’ answers about their satisfaction with information given and caretaking. Interestingly, complaints about the mask fit are very rare for conventional masks, while patients with stereotactic high-precision masks complain more often [23].

This paper attempts to compare the intra-fractional fixation accuracy of conventional stereotactic high-precision radiotherapy masks by Brainlab AG (further on abbreviated with SRTM) to novel 3D-printed masks (further on abbreviated with 3DPM) in four healthy volunteers simulating compliant and noncompliant patient behavior.

## Methods

### Design and manufacturing of the masks and fixation system

**Fig. 1:**
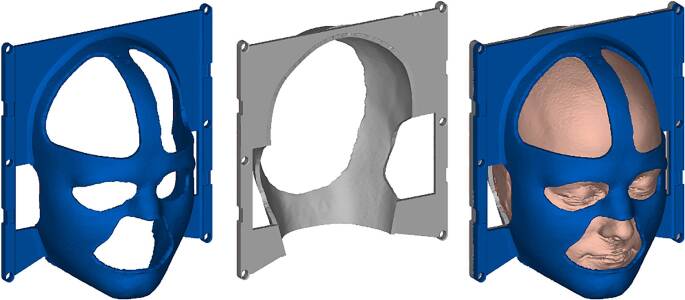
Virtual design of the 3D-printed mask system

Conventional stereotactic high-precision radiotherapy masks (SRTM) from Brainlab AG consisting of three plates were thermoformed on the heads of the four healthy volunteers (two male, two female) following the standard procedure used in the hospital. For the design of the 3D-printed masks (3DPM), MRI data of the same volunteers’ heads were recorded, segmented, and transformed to a virtual shell model in the Mimics® software (Materialise, Leuven, Belgium). A thickness of 4 mm was given using 3‑matic® (Materialise). Table fixations and cutouts for eyes, mouth, nose, ears, and hair were designed in NX (Siemens AG, Munich, Germany), using templates for faster design (Fig. 1). After evaluating the print accuracy, the masks were additively manufactured on the extrusion-based 3D printer Evolizer (EVO-tech GmbH, Schörfling, Austria). As material, acrylonitrile butadiene styrene (ABS), a standard 3D-printing filament, was used, as several studies have identified it as suitable for this application, also considering the attenuation properties [14, 20, 24, 25].

**Fig. 2:**
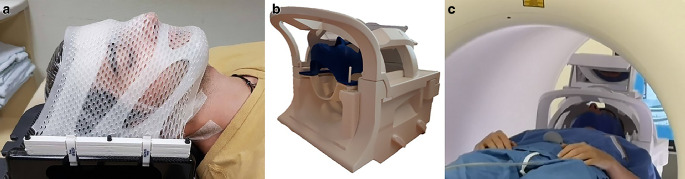
Conventional high-precision mask for fractionated stereotactic radiotherapy from Brainlab (Munich, Germany); **a**); magnetic resonance imaging (MRI) head coil with the designed fixation device and 3D-printed mask (**b**); volunteer lying in the MRI with the 3D-printed mask for the measurement (**c**)

Because the conventional mount for the Brainlab masks did not fit into the MRI head coil and additionally contained metal parts (not allowed in MRI), a special mount was designed to fulfil these targets. For this, the surface of the head coil was scanned with a Steinbichler 3D scanner and the existing Brainlab mount for radiotherapy was measured. Based on the two geometries, a 3D-printable mount consisting only of plastic (ABS) was designed and manufactured. The Brainlab fixation had six rods for the positioning of the masks. In the MRI mount, six plastic screws were embedded so they could be used for positioning, and fixation was performed with six nuts from the top (see Fig. 2).

### Measurement protocols for simulated intra-fractional fixation accuracy evaluation

**Fig. 3:**
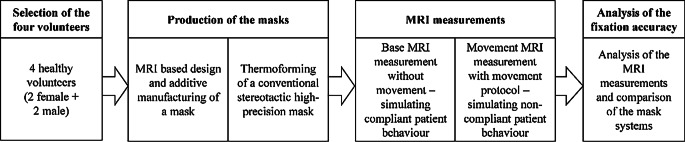
Workflow of the simulated intra-fractional fixation accuracy MRI evaluation

To have a way of objectively evaluating the movement possibility and clearance of the 3DPM compared to the SRTM, application of radiotherapy fractions was simulated. The volunteers lay in an MRI with both types of masks—once still for a base measurement, simulating compliant patient behavior, and once while following specific movement commands while their movement was tracked, simulating worst-case non-compliant patient behavior. The workflow of the study is depicted in Fig. 3.

The functional MRI’s motion-correction algorithm estimates movement based on a reference volume. Therefore, the software package Brain Voyager was used (https://www.brainvoyager.com/). For motion correction the estimated movement parameters are applied to realign the time series of brain images to the reference. The rigid body transformations for the realignment can be exported from the fMRI software. Thus, for the measurements there are three translational (X, Y, Z; in mm) and three rotational parameters (pitch, roll, yaw; in degrees) over the measurement time to compare the volunteer’s freedom of movement. The used interpolation method was a combination of trilinear for detection of motion and sinc for correction. This avoids the problem of inducing unwanted blurring effects in the data while reserving a reasonable computation time.

**Fig. 4:**
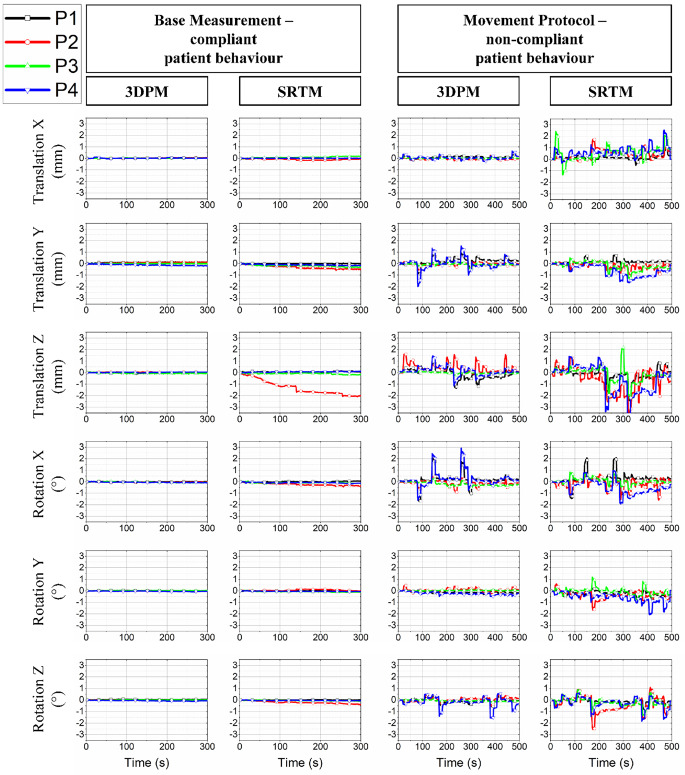
Translations and rotations in X, Y, and Z directions for the functional magnetic resonance imaging (fMRI) base and movement measurements for 3D-printed (*3DPM*) and stereotactic radiotherapy masks (*SRTM*). The base measurements simulate compliant patient behavior and the movement measurement the non-compliant patient behavior. *P* patient

To measure an equivalent to intra-fractional positioning accuracy, each volunteer underwent a functional MRI protocol for both masks, first laying still for 5 min for a base measurement while 200 datapoints with the repetition time TR = 1.5 s were collected. During the second measurement, the volunteers followed a movement instruction protocol with translation of the head in lateral (X), anteroposterior (Y), and longitudinal (Z) directions as well as rotation around these axes. These measurements took approximately 8 min with 330 datapoints recorded. An overview and schematic representation of the commands is given in Table 1. The commands were read over a mirror from a screen. Between the commands the resting position was resumed. The translational and rotational movements from the base measurements are represented in columns 1 and 2 and those from the movement protocol are represented in columns 3 and 4 in Fig. 4.

**Table 1:** Commands, parameters, and schematic of the movement protocol in the MRI measurement

Schematic	Command	Parameter
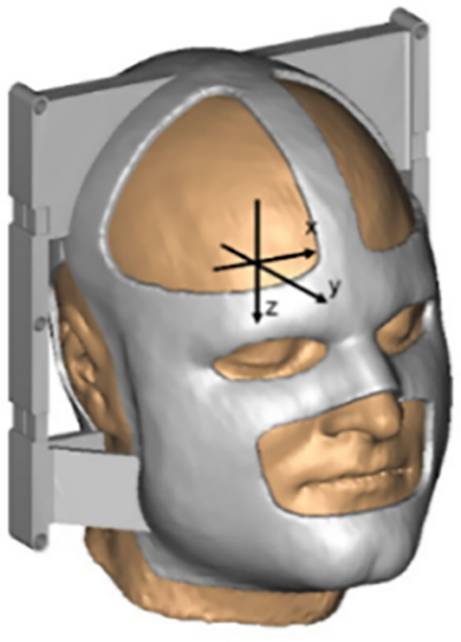	Stretch head right	Translation X
	Stretch head left	Translation X
	Stretch head forward	Translation Y
	Stretch head upwards	Translation Z
	Press chin towards chest	Pitch, rotation Z
	Press chin away from the chest	Pitch, rotation Z
	Turn nose towards right	Yaw, rotation X
	Turn nose towards left	Yaw, rotation X

### Evaluation

The translations and rotations were plotted over time, comparing for each measurement protocol the results for the four volunteers. For the base measurement, the absolute 3D displacement for each time step was calculated as the square root of the sum of the squared translations using Eq. 1. As single point values, the averaged absolute 3D displacement (Eq. 2) per volunteer and their standard deviations were also calculated. The maximum leeway per translation and rotation was derived as the absolute difference between the minimum and maximum values.

The relative translational and rotational 3D displacement, as used by Alheit et al. [1] to describe inter-fractional repositioning accuracy with Brainlab masks, is calculated as the square root of sums of the standard deviations of the translations and rotations, respectively, in all three directions (Eq. 3 and 4). To calculate the relative 3D displacement, the volunteers were not considered separately anymore but all values per translation and rotation were averaged and the standard deviation for each translational and rotational movement from all the four volunteers combined was calculated for the 3D-printed masks as well as for the conventional masks. The relative 3D displacements were then calculated using the respective standard deviations and the Eq. 3 and 4.

1 $$\textit{Absolute}\,3D\,\textit{displacement}=\sqrt{\textit{Trans}X^{2}+\textit{Trans}Y^{2}+\textit{Trans}Z^{2}}$$

2 $$\textit{Average}\,\textit{absolute}\,3D\,\textit{displacement}=\frac{\sum \sqrt{\textit{Trans}X^{2}+\textit{Trans}Y^{2}+\textit{Trans}Z^{2}}}{\textit{number}\,ofdata\,\textit{points}}$$

3 $$\textit{Relative}\,\textit{translational}\,3D\,\textit{displacement}=\sqrt{SD{\mathrm{AvgT}_{X}}^{2}+\text{SDAvg}{T_{Y}}^{2}+SD{\mathrm{AvgT}_{Z}}^{2}}$$

4 $$\textit{Relative}\,\textit{rotational}\,3D\,\textit{displacement}=\sqrt{SD{\mathrm{AvgR}_{X}}^{2}+\text{SDAvg}{R_{Y}}^{2}+\text{SDAvg}{R_{Z}}^{2}}$$

## Results and discussion

### Absolute measurement results

In Fig. 4 the absolute measurement results for translation in X, Y, and Z (T_x_, T_y_, T_z_) and rotation around X, Y, and Z (R_x_, R_y_, R_z_) are displayed over time for the base measurement and movement measurement. The diagrams show less movement and less scattering for 3DPM than for SRTM. For the measurements without movement, all volunteers show a drifting motion over time, which is to be expected due to unconscious relaxation. Volunteer P2 had a large drift with the SRTM mask during the base measurement, mostly visible in T_Z_. Notable is that the average values for T_x_, T_y_, T_z_, R_x_, R_y_, and R_z_ of each measurement lie in the range of ±1 mm and ±1° for both masks and movement protocols.

For 3DPM the leeway in all translations and rotations is consistently smaller than with the SRTM, with 3DPM:SRTM values of 0.07 mm:0.24 mm (T_x_), 0.23 mm:0.61 mm (T_y_), 0.21 mm:2.09 mm (T_z_), 0.14°:0.44° (R_x_), 0.09°:0.19° (R_y_), and 0.12°:0.40° (R_z_). Calculating the maximum 3D leeway as the square root of the sum of the squared translations for the 3DPM, the maximum 3D leeway is 0.32 mm, while it reaches 2.19 mm for the SRTM.

Less translations and rotations for 3DPM vs. SRTM are also visible in the movement measurements. For T_X_, the 3DPM allow maximally 0.05 mm, whereas for the conventional masks, the graphs vary between −1.5 mm and +2.5 mm. A similarly significant difference can be derived from the R_Y_, where the values for the 3D-printed masks also lie between ±0.5° and the values for the conventional masks vary between −2° and +1.2°. Also, for T_Z_, R_X_, and R_Z_, the range is smaller for the 3D-printed masks than for the conventional ones. Only for T_Y_ do the graphs for 3D-printed masks vary between −2 mm and +1.5 mm, while the graphs for the conventional masks vary only between −1.5 mm and +0.8 mm.

For the volunteer P1, both masks allow most movement for T_Z_ and R_X_ and both masks seem to fix the volunteer quite well. The intended big movements are clearly visible in the peaks of the graphs for SRTM and 3DPM. T_X_ and R_Y_ are higher for SRTM; for the other translations and rotations, no significant differences between the conventional and the 3D-printed mask are visible.

For the volunteer P2, the baseline for the SRTM is quite unsteady. It shows a drift away from the original position over time. The intended movements following the protocol for the conventional and the 3D-printed masks do not have overlapping peaks. Thus, it is hard to compare the allowed movements in the masks per orientation. However, overall, the graphs indicate that the conventional mask allows more movement than the 3D-printed mask. The largest movements with the 3D-printed mask are in T_Z_.

For the volunteer P3, in the base measurements there is a drift up until +0.2 mm in T_X_ and −0.4 mm in T_Y_ during the base measurement with SRTM, which is not noticeable with 3DPM. The difference in fixation accuracy comes out very clearly by comparing the movement measurements. The conventional mask allows movements between −1.4 mm and +2.5 mm for T_X_ and ±2.3 for T_Z_, which far exceeds the equivalent movements for 3DPM.

For the volunteer P4, the differences in the peaks for the movements are visible for both masks. SRTM allows more movements in T_X_, T_Z_, and R_Y_, while in T_Y_ and R_X_, the 3DPM shows higher amplitudes. R_Z_ is nearly equal for both masks, with a slightly larger amplitude at the last intended movement.

### Absolute 3D displacement over time per volunteer

**Fig. 5:**
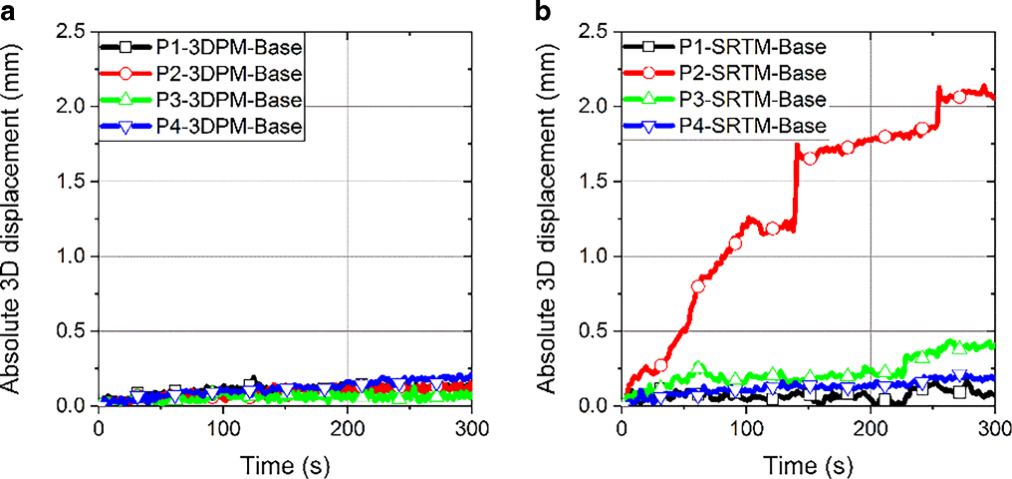
Absolute 3D displacements over time for base measurements with the 3D-printed mask (*3DPM*); **a**) and the stereotactic radiotherapy mask (*SRTM*); **b**) calculated with Eq. 1. *P* patient

To bring these graphs into a more compact format, the absolute 3D displacements over time were calculated for the base measurements with Eq. 1 and are plotted in Fig. 5. While the four graphs for 3DPM are close together, with absolute displacement values lower than 0.25 mm, the graphs of the measurements with the conventional SRTM masks are more spread out and for the volunteer P2, even show values above 2 mm. This is mainly caused by the influence of their movement in the Z direction. Measurements with both types of masks show that the volunteers’ heads shifted with time unconsciously. This is to be expected also during fractions, as the patients relax slightly in their position over time. These measurements indicate that this shift is less pronounced when wearing the 3D-printed mask.

### Average absolute 3D displacement per volunteer

**Fig. 6:**
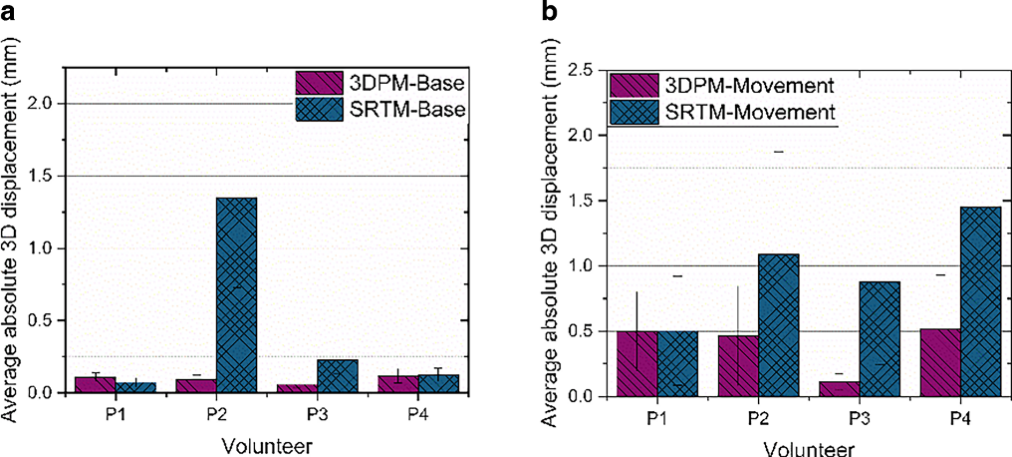
Average absolute 3D displacement with standard deviations with the 3D-printed mask (*3DPM*) and the stereotactic radiotherapy mask (*SRTM*) for base measurement (**a**) and measurement with movement protocol (**b**) calculated with Eq. 2. *P* patient

To reduce the results to single point values per volunteer and measurement, the average absolute 3D displacements and their respective standard deviations are calculated using Eq. 2 (see Fig. 6). For three volunteers, the average absolute 3D displacement with 3DPM is lower than with SRTM; only for volunteer P1 did the SRTM show a better fixation accuracy during the base measurement and the same fixation accuracy with both masks for the movement measurement. For the base measurements, the results were P1:0.11:0.07; P2:0.09:1.35; P3:0.06:0.23; and P4:0.12:0.13 (volunteer:average absolute displacement 3DPM [mm]:average absolute displacement SRTM [mm]). For the movement measurement the results were P1:0.50:0.50; P2:0.46:1.09; P3:0.11:0.88; and P4:0.51:1.45. At the base measurements, the greatest difference between average absolute 3D displacements per mask are visible for P2 and P3. For the movement measurement, the difference is obvious for P2, P3, and P4. Most obvious are the large standard deviations for SRTM, indicating a lot of movement, and the large displacement of P2 at the base measurement, due to the large unconscious drift.

### Relative 3D displacement

For the base measurements, 3DPM had a relative translational 3D displacement of 0.10 mm compared to SRTM which allowed 0.67 mm. For the relative rotational 3D displacement, with 0.06°, the 3DPM also allowed less rotation than the conventional mask with 0.15°. With active movement the values for the relative 3D displacements were 0.56 mm and 0.63° for 3DPM compared to 1.01 mm and 0.77° for SRTM.

## Conclusion and outlook

The biggest advantage of 3D-printed masks compared to stereotactic high-precision masks lies in the design that allows eyes, mouth, nose, and ears to be uncovered by material. This allows free breathing and view for the patients, thus possibly creating higher comfort during an already stressful time. As the long 1‑hour conventional process of thermoforming the masks on the patients’ faces was reported to be the most distressing part of radiation therapy, it is safe to say that by replacing this by a design and manufacturing process in the absence of the patient, patient comfort can be raised. Additive manufacturing allows patient-specific parts to be manufactured in the absence of the patient. All that is necessary to create a virtual model of a patient fixation mask is MRI or CT data, software, and knowledge and experience.

3D-printed fixation masks are especially beneficial for pediatric patients, as current masks are not adapted for children [18] and many children need general anesthesia for a head support placement [12]. The design freedom allows creation of nicer (less frightening) designs for children, e.g., a mask in Mickey Mouse design or in the patient’s favorite color.

Our study focused on stereotactic high-precision masks because these are more expensive than standard thermoplastic masks and 3DPMs are more likely to be economically competitive with these kinds of masks. However, obviously the demands for fixation accuracy for the high-precision masks are higher. With this study, we could show that our design of masks reaches at least the same intra-fractional fixation accuracy as with SRTMs and in most cases even achieves better fixation accuracy.

The price for 3D-printed masks is competitive with that of stereotactic high-precision masks, with variable costs being even lower for the 3D-printed masks. In this case, 3DPM had variable costs of 71 € vs. 118 € for SRTM, including labor (technician/nurse), material, and machine costs [23]. With the automatization of the design procedure, further cost reduction or at least faster production time can be expected with higher reproducibility of the design outcome.

Analyzing the functional MRI measurements, the 3D-printed masks show fixation accuracy in the range of or better than the conventional SRTM masks. While the absolute average values for the translations and rotations lie in the same range—between ±1 mm and ±1° for both masks—the standard deviations and the relative and absolute 3D displacements tend to be lower for 3DPM than for SRTM. Therefore, the intra-fractional fixation accuracy should be good enough for clinical practice, keeping in mind that the study has the limitation of only containing four, healthy volunteers.

Inter-fractional shifting values are reportedly higher than intra-fractional shifting values when comparing the same mask systems [20]. Even though best practice in radiotherapy is to use image-guiding systems to correct for set-up errors, as a next step, the inter-fractional positioning accuracy could be assessed. Even though the inter-fractional set-up accuracy of 3D-printed masks was studied by Haefner et al. (2018) and found to be sufficient [20], another study with our design is recommended. The set-up accuracy of our design is expected to be even better than that achieved with the designs of Haefner et al. (2018) [20], because our design is meant to reach the accuracy of stereotactic high-precision masks and, therefore, does not only consist of a patient-specific frontal part of the mask, but also has a back part of the mask that fits to the patient’s head, while Haefner et al. (2018) use a head rest that is reusable for multiple patients [20]. When 3D-printed masks become standard practice in hospitals and can be used instead of standard thermoplastic masks, a reusable head rest would, however, save costs and could be an interesting addition.
